# Supplementary material for: Impact of household economic strengthening intervention on food security among caregivers of orphans and vulnerable children in Tanzania
Source: PLoS One. 2022 Feb 25;17(2):e0264315. doi: 10.1371/journal.pone.0264315 (PMC8880745; doi:10.1371/journal.pone.0264315)
Supplement: Attachment — Submitted filename: Response to Reviewers.docx [file pone.0264315.s006.docx]

**RESPONSES TO REVIEWERS’ COMMENTS**

PONE-D-21-06461
Impact of household economic strengthening intervention on food security among caregivers of orphans and vulnerable children in Tanzania
PLOS ONE

| **Academic Editor’s comments** | **Authors’ response** |
| --- | --- |
| Please use an existing conceptual framework from literature or develop your own conceptual framework to guide your multivariate modelling. Otherwise, it seems a data mining exercise! | Thank you.  As advised, we developed a conceptual framework to guide our multivariate modeling. See lines 259–279, and Fig 1. |
| Journal Requirements:  When submitting your revision, we need you to address these additional requirements.    1. Please ensure that your manuscript meets PLOS ONE's style requirements, including those for file naming. The PLOS ONE style templates can be found at  <https://journals.plos.org/plosone/s/file?id=wjVg/PLOSOne_formatting_sample_main_body.pdf> and  [https://journals.plos.org/plosone/s/file?id=ba62/PLOSOne_formatting_sample_title_authors_affiliations.pdf](about:blank) | Thank you.  We have adhered to all PLOS ONE requirements, including file naming. |
| 2. Please include additional information regarding the survey or questionnaire used in the study and ensure that you have provided sufficient details that others could replicate the analyses. For instance, if you developed a questionnaire as part of this study and it is not under a copyright more restrictive than CC-BY, please include a copy, in both the original language and English, as Supporting Information. | Thank you.  As this was a secondary analysis of existing program data, there was no tools developed specifically for this study. However, the tools used for data collection at baseline (i.e. during screening and enrollment of beneficiaries into the USAID Kizazi Kipya project) and midline are described under “Study design and data collection tools” section of the manuscript. See lines 174–183, and 204-210. |

| **Reviewers’ comments** | **Authors’ response** |
| --- | --- |
| Reviewer's Responses to Questions  Comments to the Author  1. Is the manuscript technically sound, and do the data support the conclusions?  The manuscript must describe a technically sound piece of scientific research with data that supports the conclusions. Experiments must have been conducted rigorously, with appropriate controls, replication, and sample sizes. The conclusions must be drawn appropriately based on the data presented.  Reviewer #1: Yes  Reviewer #2: Partly | Thank you.  We have revised the manuscript to improve all areas where changes were asked. We believe that the current version of the manuscript is technically and scientifically sound. |
| 2. Has the statistical analysis been performed appropriately and rigorously?  Reviewer #1: Yes  Reviewer #2: No | Thank you.  We believe the statistical analysis employed in this study was appropriate and rigorous. We have provided clarifications regarding how the analysis was performed and why we chose to use those statistical techniques. See lines 244–247. |
| 3. Have the authors made all data underlying the findings in their manuscript fully available?  The PLOS Data policy requires authors to make all data underlying the findings described in their manuscript fully available without restriction, with rare exception (please refer to the Data Availability Statement in the manuscript PDF file). The data should be provided as part of the manuscript or its supporting information, or deposited to a public repository. For example, in addition to summary statistics, the data points behind means, medians and variance measures should be available. If there are restrictions on publicly sharing data—e.g. participant privacy or use of data from a third party—those must be specified.  Reviewer #1: Yes  Reviewer #2: No | Thank you.  The data supporting our results and conclusions were all provided in figures and tables as part of the manuscript. In addition, we have provided anonymous data files for each of the tables and figures presented, see “Supporting Information” section, lines 751–756, as well as the actual data files uploaded. |
| 4. Is the manuscript presented in an intelligible fashion and written in standard English?  PLOS ONE does not copyedit accepted manuscripts, so the language in submitted articles must be clear, correct, and unambiguous. Any typographical or grammatical errors should be corrected at revision, so please note any specific errors here.  Reviewer #1: Yes  Reviewer #2: Yes | Thank you. |
| 5. Review Comments to the Author  **Reviewer #1:** |  |
| I read with great interest the paper. I find it well wrote on important focus and setting  below my suggestions  1. Introduction: Use the concept of children at risk to define your vulnerable population. The concept of “children at risk” changes worldwide according to each specific context. Africa has a large burden of overall risk factors related to childhood health and development, most of which are of an infective or social origin (The At Risk Child Clinic (ARCC): 3 Years of Health Activities in Support of the Most Vulnerable Children in Beira, Mozambique. Int J Environ Res Public Health). In fact, In the African continent, the greatest risks to children’s health and development are mainly of infective and social origin, particularly exposure to Human Immunodeficiency Virus (HIV), malaria, TB, and malnutrition. It is no coincidence that the scope for child development improvement at the regional level can be identified within three domains: nutrition, environment, and mother-child interaction. | Thank you.  The study population is appropriately defined under the study population section (lines 185–195). We have also added a few lines in the introduction to state that the caregivers are taking care of children who are orphans or vulnerable due to HIV. They are from households that are HIV affected or infected (lines 99–101). |
| 2. Methods and results are clear | Thank you. |
| 3. Discussion: add the role of social determinant of health and discuss better this aspect.  Give some take home message that came from your interesting paper on global health approach. | Thank you.  We have revised the Discussion section to describe food security and the factors affecting it in the context of social determinants of health (SDH). Takeaway messages are included for the key factors discussed. See the “Discussion” section, lines 407 – 527. |
| Reviewer #2: |  |
| 1. The data analysis and multivariate modelings needs a major review to include more data/information. For instance in Table 3, (n=132,583) is the number of households at baseline, yet in the three models the n=265,166. How did the authors come by that number (365,166) at mid-line? The number at follow-up cannot be more than the number at baseline, else there may be no basis to measure change/impact. Something may be missing here, how many households were interviewed at mid-line and were there the same households interviewed at baseline? Can the authors show the numbers also in variables? Also, we should be comparing the food security situation between those in the intervention (WORTH Yetu) and those who are not in the intervention to identify improvement and not those withing the same group. I recognize that in Figure 1(appendix) there is a comparison of baseline and mid-line with same numbers. But this is not clear because the authors indicated that at mid-line only 10,123 out of the 132,583 households belong to the intervention. | Thank you.  We have further clarified that since food security was measured twice – at baseline and at midline – for each caregiver, there were 132,583*2 = 265,166 total observations for which each multivariate model was conducted (see lines 244–246). Also see the footnotes of Table 2 (the former Table 3).  Note that there was one caregiver for each household. Therefore, the 132,583 as the number of caregivers is equally the number of households for which food security was assessed at baseline and at midline. So, the baseline and midline households were the same; that is, midline data came from the same households surveyed at baseline.  Absolute numbers in the variables and their categories are shown in Table 1. The numbers are also indicated in respective titles of the tables and figures.  The comparison of food security situation between those in the intervention (WORTH Yetu) and those who are not in the intervention is clearly presented in Fig 4 and described in the main text of the manuscript, lines 337–344. The figure also shows how the food security situation vary by other background characteristics, lines 346–366. |
| 2. Some of the interpretations of the results in Table 3 are wrong. For instance, the interpretation of the Odds Ratios for Rural Vs Urban dwellers (Line 348 - 350) is wrong, it should be the opposite. "While caregivers in rural areas were less likely to be in little to no hunger households, they were more likely to be in both moderate and severe hunger households, than their urban counterparts". They authors however interpreted it correctly in the discussion section (lines 427-430 | Thank you.  We have corrected the sentence (see lines 396–398). |
| 3. There are Tables 1 and 3, but no table 2 is shown throughout the manuscript | Thank you.  We have reworked the numbering, considering that since there was no Table 2, the then Table 3 is now Table 2. So, Table 3 is no longer existing (see lines 369, 387, 391 (table title), and 393). |
| 4. Table 1 showed be presented including the data of the mid-line. That is, show baseline and Mid-line side-by-side. | Thank you.  As the characteristics of the respondents (Table 1) were largely similar at baseline and at midline (i.e. there was no many years apart between the surveys), we retained the baseline characteristics. |
| 5. The Authors indicated the study design is longitudinal: baseline data in 2017/18 and mid-line data in 2019. When exact (month) did the baseline end? and when exactly (period/month) in 2019 was the mid-line data collection done? This information is important because it has implication for the results and conclusions drawn.  We are told the intervention began in 2019, when will the intervention end? | Thank you.  As advised, we have provided more information regarding the start and end dates of both the baseline and midline (see “The study design and data collection tools” section, lines 174–183. In addition, we have also provided the duration regarding how long each caregiver had been in the project from baseline to midline (see lines 310 – 316, and Fig 2).  The intervention will continue until the end of the project on 30th September 2021. |
| 5. The authors should provide more details about the study design and data collection including number interviewed at mid line etc. | Thank you.  We have provided more details about the study design and data collection. The number of caregivers interviewed and the number of households they were from are indicated; see lines 174–195. |
| 6. PLOS authors have the option to publish the peer review history of their article (what does this mean?). If published, this will include your full peer review and any attached files.  If you choose “no”, your identity will remain anonymous but your review may still be made public.  Do you want your identity to be public for this peer review? For information about this choice, including consent withdrawal, please see our Privacy Policy.  Reviewer #1: Yes: Di Gennaro Francesco  Reviewer #2: No | Thank you. |

**END**
